# Combining CRP and CA19-9 in a novel prognostic score in pancreatic ductal adenocarcinoma

**DOI:** 10.1038/s41598-020-80778-0

**Published:** 2021-01-12

**Authors:** Anna M. Nurmi, Harri K. Mustonen, Ulf-Håkan Stenman, Hanna E. Seppänen, Caj H. Haglund

**Affiliations:** 1 Department of Surgery, Helsinki University Hospital, University of Helsinki, PO Box 22, 00014 Helsinki, Finland; 2 Department of Clinical Chemistry, Helsinki University Hospital, Haartmaninkatu 8, PO Box 63, 00014 Helsinki, Finland; 3 Translational Cancer Medicine Research Program, Faculty of Medicine, University of Helsinki, Haartmaninkatu 8, PO Box 63, 00014 Helsinki, Finland

**Keywords:** Pancreatic cancer, Tumour biomarkers

## Abstract

Inflammation promotes tumor progression, induces invasion and metastatic spread. This retrospective study explored CRP, CA19-9, and routine laboratory values as preoperative prognostic factors in pancreatic cancer patients. Between 2000 and 2016, there were 212 surgically treated pancreatic cancer patients at Helsinki University Hospital, Finland. Out of these, 76 borderline resectable patients were treated with neoadjuvant therapy (NAT); 136 upfront resected patients were matched for age and sex at a 1:2 ratio. We analyzed preoperative CRP, CA19-9, CEA, leukocytes, albumin, bilirubin and platelets. CRP and CA19-9 were combined into a prognostic score: both CRP and CA19-9 below the cut-off values (3 mg/l and 37 kU/l, respectively), either CRP or CA19-9 above the cut-off value, and finally, both CRP and CA19-9 above the cut-off values. Among all patients, median disease-specific survival times were 54, 27 and 16 months, respectively (*p* < 0.001). At 5 years, among patients with CRP and CA19-9 levels below the cut-off values, 49% were alive and 45% were disease-free. Among NAT patients the corresponding survival rates were 52% and 45% and among those undergoing upfront surgery 45% and 40%, respectively. This novel prognostic score combining CRP and CA19-9 serves as a useful preoperative tool estimating survival.

## Introduction

Pancreatic ductal adenocarcinoma (PDAC) carries one of the worst survival rates among all cancers. With a 5-year overall survival of less than 10%^1^, surgery combined with oncological therapy offers the only potential cure. However, only 15–20% of all patients are considered resectable^2^ and even among resected patients, the 5-year survival rate remains poor, at 20–25%^2,3^. The median survival for upfront resectable PDAC patients has previously been reported as stage-dependent: ranging from 14 (Stage III) to 46 (Stage IA) months^2,4^. Neoadjuvant therapy (NAT) appears to improve survival in patients with advanced disease^5,6^ and a median survival of 20–35 months has been reported depending upon the study inclusion criteria, resection rate and the treatment regimens used^6–9^.

CA19-9 is a sialylated Lewis blood group antigen and it is the most commonly used biomarker for PDAC. CA19-9 levels correlate with tumor burden and disease progression, and in both non-resectable and resectable patients, CA19-9 normalization during NAT associates with a better prognosis^10^. One limitation of CA19-9 is that 5–10% of the Caucasian population are Lewis antigen-negative and cannot synthesize CA19-9, possibly resulting in normal levels even in advanced PDAC^11^.

The cancer-related inflammatory response, which can be divided into local and systemic^12^, appears to further promote tumor progression, increase angiogenesis and local immunosuppression^13–15^. As a part of the systemic inflammatory response, C-reactive protein (CRP) secretion is increased and albumin secretion decreased^16^. Both elevated CRP and tumor marker levels represent indicators of a poor prognosis in resected PDAC patients^17^. However, in pancreatic cancer studies, the cut-off values for CRP have varied and many studies involved only small-scale patient samples or only patients with advanced disease^18–24^. Furthermore, we lack data on CRP levels in patients treated with NAT and subsequent surgery. No universal prognostic factors exist for patients treated with NAT, although there is evidence that NAT affects the tumor, the tumor microenvironment and the cancer-related inflammatory response^25^. In addition, most prognostic factors are available only after surgery.

This study aimed to explore the predictive value of CRP, CA19-9, and routine preoperative laboratory tests among surgically treated PDAC patients. We compared patients treated with NAT and subsequent surgery to patients undergoing upfront surgery. Based on current knowledge that NAT affects the inflammatory response and tumor burden, we hypothesized that these markers would impact survival differently in patients treated with NAT and those undergoing upfront surgery.

## Material and methods

### Patients

Between 2000 and 2016, in total 94 borderline resectable pancreatic cancer patients were treated with NAT and subsequent surgery at Helsinki University Hospital, Finland. These were compared with 182 resectable patients who underwent upfront surgery. Patients undergoing upfront surgery were matched for age and sex at a 1:2 ratio. We excluded patients without preoperatively collected plasma samples (n = 47), those receiving oral immunosuppressive medication, patients with an ongoing infection at the time of surgery or undergoing emergency surgery and those who died from surgery-related complications (n = 17). Patient characteristics and survival data were retrospectively collected from a prospectively maintained database using patient records and the Finnish Population Registry. Statistics Finland provided death certificates. All histological samples were re-evaluated by an experienced pathologist to confirm the PDAC diagnosis. The study was conducted in accordance with the principles of the Declaration of Helsinki and its later amendments. The Surgical Ethics Committee of Helsinki University Hospital (226/E6/2006, extension 4/17/2013, extension 3/27/2019) and the National Supervisory Authority of Welfare and Health approved this study. All research was performed in accordance with relevant regulations and all patients signed a written informed consent form agreeing to their blood samples and data collected and used for research purposes.

### Neoadjuvant therapy

NAT consisted of FOLFIRINOX, gemcitabine alone or in combination with capecitabine, cisplatin or nab-paclitaxel. Additional radiotherapy was administered to 31 (35%) patients. NAT was administered to borderline resectable patients, defined as superior mesenteric vein, portal vein or superior mesenteric artery contact upon diagnosis. Supplementary Table 1 describes in detail the treatment regimens along with the administered adjuvant therapy regimens.

**Table 1:** Cut-off values with the number of patients and comparison of median values between NAT patients and those undergoing upfront surgery.

	NAT n = 76 (%)	US n = 136 (%)	*p *value	Median for NAT patients (IQR)	Median for US patients (IQR)	*p *value
**CRP (mg/l)**						
< 3	40 (53)	65 (48)	0.567	2.7 (1.2–5.9)	3.0 (1.5–8.6)	0.246
≥ 3	36 (47)	71 (52)				
**CA19-9 (kU/l)**						
≤ 37	31 (41)	43 (32)	0.229	91 (15–373)	125 (22–667)	0.287
> 37	45 (59)	93 (68)				
**CRP and CA19-9**						
CRP < 3 and CA19-9 ≤ 37	19 (25)	20 (15)	0.189	–	–	
CRP < 3 and CA19-9 > 37 OR CRP ≥ 3 and CA19-9 ≤ 37	33 (43)	68 (50)				
CRP ≥ 3 and CA19-9 > 37	24 (32)	48 (35)				
**Albumin (g/l)**						
< 35	15 (20)	28 (21)	1.000	38.0 (35.9–39.9)	38.0 (35.2–40.4)	0.770
≥ 35	60 (79)	108 (79)				
Missing	1 (1)	0				
**CEA (µg/l)**						
≤ 5.0	65 (86)	106 (78)	0.195	2.7 (1.5–4.0)	2.9 (1.7–4.5)	0.430
> 5.0	10 (13)	29 (21)				
Missing	1 (1)	1 (1)				
**Bilirubin (μmol/l)**						
≤ 20	73 (96)	87 (64)	** < 0.001**	8 (6–11)	15 (9–32)	** < 0.001**
> 20	2 (3)	49 (36)				
Missing	1 (1)	0				
**Platelets (E9/l)**						
< 150	6 (8)	11 (8)	0.489	220 (192–276)	241 (195–289)	0.279
150–360	61 (80)	115 (85)				
> 360	9 (12)	10 (7)				
**Leukocytes (E9/l)**						
< 3.4	5 (7)	2 (1)	0.194	5.9 (4.8–7.3)	6.1 (5.4–7.8)	0.068
3.4–8.2	59 (78)	108 (79)				
> 8.2	12 (16)	26 (20)				
**GPS score (CRP mg/l, albumin g/l)**						
0 (CRP ≤ 10 and albumin ≥ 35)	65 (85)	107 (79)	0.281	–	–	
1 (CRP > 10 or albumin < 35)	5 (7)	17 (12)				
2 (CRP > 10 and albumin < 35)	5 (7)	12 (9)				
Missing	1 (1)	0				
**mGPS score (CRP mg/l, albumin g/l)**						
0 (CRP ≤ 10)	55 (72)	91 (67)	0.366	–	–	
1 (CRP > 10)	15 (20)	33 (24)				
2 (CRP > 10 and albumin < 35)	5 (7)	12 (9)				
Missing	1 (1)	0				
**DSS (months, from surgery)**						
< 12	15 (20)	32 (24)	0.338	–	–	
12–24	15 (20)	32 (24)				
> 24	46 (60)	72 (52)				
**DFS (months, from surgery)**						
< 12	37 (49)	67 (49)	0.868	–	–	
12–24	16 (21)	32 (24)				
> 24	23 (30)	37 (27)				

We used the manufacturer’s recommended cut-off values for CRP, CEA, platelets, bilirubin, and leukocytes, and for CA19-9 based on the literature^26^ and for albumin as in the Glasgow prognostic score^27^. For the Glasgow prognostic score (GPS)^27^ and the modified Glasgow prognostic score (mGPS)^28^, cut-off values for CRP and albumin are based on literature.

*IQR* interquartile range, *NAT* neoadjuvant therapy, *US* upfront surgery.

### High-sensitivity CRP and other laboratory values

CRP was determined from preoperatively collected plasma samples (n = 212). Samples were stored at − 80 °C until assayed at our research laboratory at the University of Helsinki. A high-sensitivity CRP method was used given its ability to measure lower levels of circulating CRP than possible with standard methods. A monoclonal antibody (anti-hCRP, code 6405, Medix Biochemica, Espoo, Finland) was used to capture CRP and as a tracer in a sandwich assay, a method described in more detail before^17^. Other preoperative laboratory values consisted of routine laboratory tests analyzed at the clinical laboratory of Helsinki University Hospital: albumin (g/l, n = 211), CA19-9 (kU/l, n = 212), CEA (μg/l, n = 210), leukocytes (E9/l, n = 212), platelets (E9/l, n = 212) and bilirubin (μmol/l, n = 211). Samples for CRP (96%, n = 204) and routine laboratory tests (97%, n = 206) were primarily collected 1–4 days preoperatively. Tumor marker levels were in 90% of patients (n = 191) determined 1–7 days preoperatively (range 1–60 days). Among NAT patients, all samples were collected following NAT. Table 1 lists the cut-off values along with a comparison of the median values between NAT patients and those undergoing upfront surgery. For laboratory values, we used the manufacturer’s recommended cut-off values for CEA, platelets, bilirubin, and leukocytes, for CA19-9 based on the literature^26^, and for albumin based on the Glasgow prognostic score (GPS)^27^. For GPS and the modified Glasgow prognostic score (mGPS)^28^, cut-off values were based on the literature. The cut-off value for CRP (3 mg/l) was based on the cut-off value used in Finland. We explored higher cut-off values for CRP, finding that they did not differentiate survivors as well as using 3 mg/l as the cut-off value (data not shown).

### Statistics

We tested the distribution of continuous variables for normality using the Kolmogorov–Smirnov test. To compare laboratory values between groups, the Mann–Whitney U and Jonckheere–Terpstra tests were used for continuous variables. The Fisher’s exact test and linear-by-linear association were used for categorical variables. We estimated survival using the Kaplan–Meier method (log rank). Primary end-point was disease-specific survival (DSS) which was determined from surgery to cancer-specific death. Secondary end-point was disease-free survival (DFS), which was determined from surgery to the first recorded disease progression or death from PDAC. We calculated multivariate analyses using the Cox proportional hazards method: age, sex, stage, tumor grade, adjuvant therapy, perivascular invasion, resection margin status, albumin, and CRP and CA19-9 were considered clinically relevant and included in the model. Variables were examined for possible interactions. For each variable, the assumption of a constant proportional hazard rate over time was tested using a time-dependent variable; all variables except tumor grade met the assumption. Thus, the model was stratified by tumor grade. Additionally, the area under the receiver operating characteristic (ROC) curve at 5 years postoperatively was calculated. Patients with missing data were censored from survival analyses. The end of follow-up was January 14, 2020, with a minimum follow-up of 3 years or until death. We calculated all statistical analyses with SPSS (version 24, IBM, New York, NY, USA), and considered *p* < 0.05 as statistically significant. All tests were two-tailed.

## Results

After exclusions, 76 patients were treated with NAT and subsequent surgery and 136 patients underwent upfront surgery (total n = 212). The median follow-up time was 2.2 years. Table 2 shows the comparison of clinicopathological characteristics between NAT patients and those undergoing upfront surgery.

**Table 2:** Clinicopathological comparison between NAT patients and those undergoing upfront surgery.

	NAT, n = 76 (%)	US, n = 136 (%)	*p *value
**Age at operation, median (range)**	65.5 (44.6–82.5)	64.9 (44.6–80.0)	0.761
≥ 65 years	39 (51)	67 (49)	0.886
**Gender, male**	33 (43)	59 (43)	1.000
pTN (AJCC 8th edition)*, T0	2 (3)	0	**0.002**
T1	20 (26)	18 (13)	
T2	47 (62)	93 (68)	
T3	7 (9)	25 (19)	
T4	0	0	
N0	37 (49)	38 (28)	**0.004**
N1	26 (34)	59 (43)	
N2	13 (17)	39 (29)	
Stage (AJCC 8th edition)*, 0	1 (1)	0	**0.001**
IA	10 (13)	11 (8)	
IB	25 (34)	24 (18)	
IIA	1 (1)	3 (2)	
IIB	26 (34)	59 (43)	
III	13 (17)	39 (29)	
Grade*, 1	13 (17)	24 (18)	0.504
2	45 (59)	81 (60)	
3	14 (19)	28 (20)	
Missing	4 (5)	3 (2)	
**pTumor size (mm), median (IQR)**	25 (20–35)	30 (25–40)	**0.011**
Tumor size, ≤ 30 mm	56 (74)	75 (55)	**0.008**
**R0 resection**	57 (77)	99 (77)	1.000
**Vascular resection**	41 (54)	40 (29)	**0.001**
**Perineural invasion**	53 (70)	101 (76)	0.333
**Perivascular invasion**	24 (32)	49 (38)	0.451
**Adjuvant therapy**	52 (68)	94 (69)	0.820
Discontinuation	14 (27)	30 (32)	0.576
**Survival,** DSS	28 (23–32)	26 (21–31)	0.795
DFS	12 (11–14)	12 (9–14)	0.843

Missing data: Information on perineural and perivascular invasion was missing in 6 patients. Resection margin status was missing in 9 patients. No tumor grade was available for 7 patients due to a complete response or inconclusive data. The Fisher’s exact and linear-by-linear association (*) were used for categorical variables, while the Mann–Whitney U test was used for continuous variables.

*AJCC* American Joint Committee on Cancer, *IQR* interquartile range, *NAT* neoadjuvant therapy, *US* upfront surgery.

### Combining CRP and CA19-9 in a prognostic score

**Figure 1:**
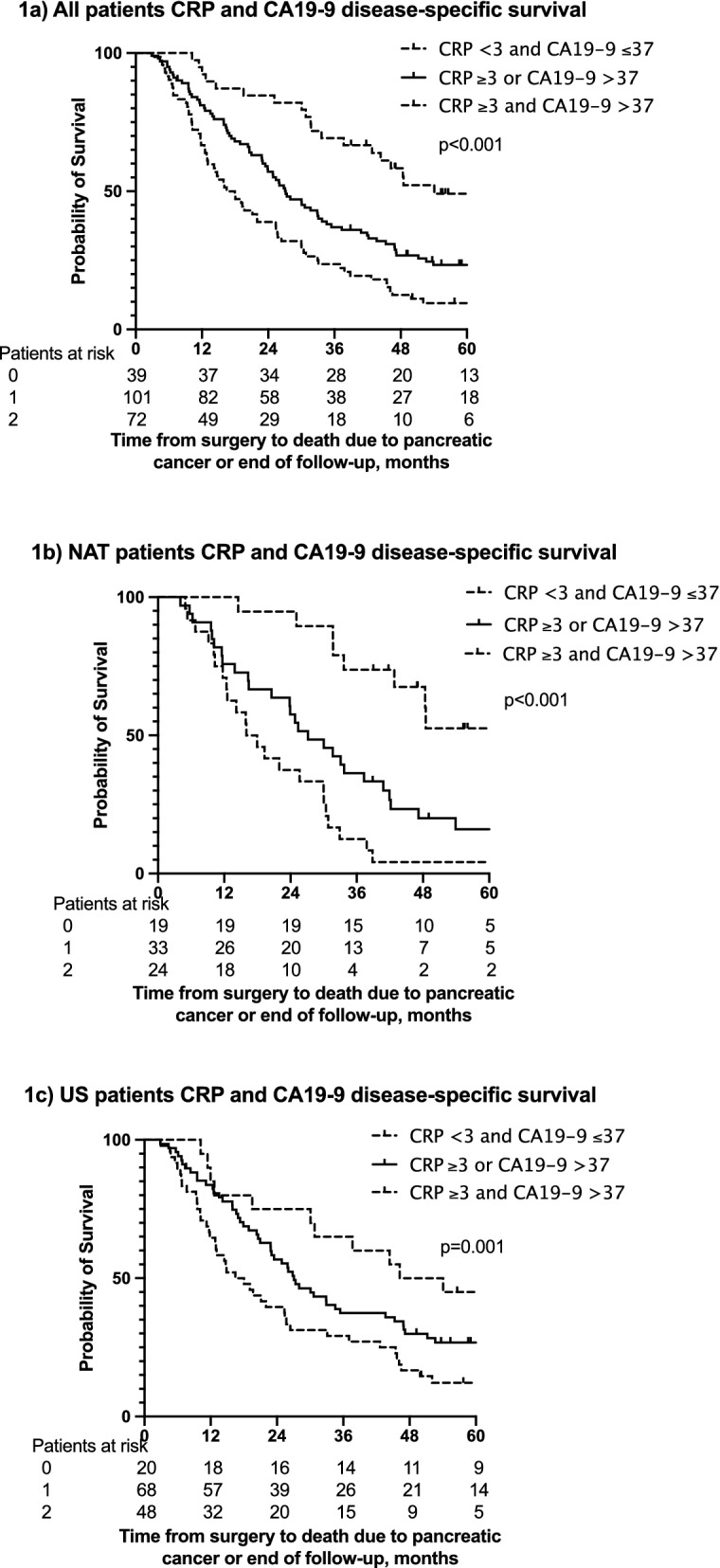
(**a**) Disease-specific survival (DSS) for all patients according to CRP and CA19-9 levels. Median DSS were 54 months (m) (95% CI –), 27 m (95% CI 22–32 m), and 16 m (95% CI 12–21 m). The area under the receiver operation characteristic (ROC) curve at 5 years was 0.686 (95% CI 0.597–0.776) and the overall concordance score was 0.690 (95% CI 0.636–0.745). (**b**) DSS for NAT patients according to CRP and CA19-9 levels. The median DSS for NAT patients with low CRP and CA19-9 has not been reached yet. Median survival for those with either above the cut-off value was 27 m (95% CI 18–36 m) and for those with both above the cut-off value 16 m (95% CI 10–22 m). (**c**) DSS for US patients according to CRP and CA19-9 levels. The median DSS were 46 m (95% CI 25–67 m), 27 m (95% CI 21–33 m), and 16 m (95% CI 11–22 m).

**Figure 2:**
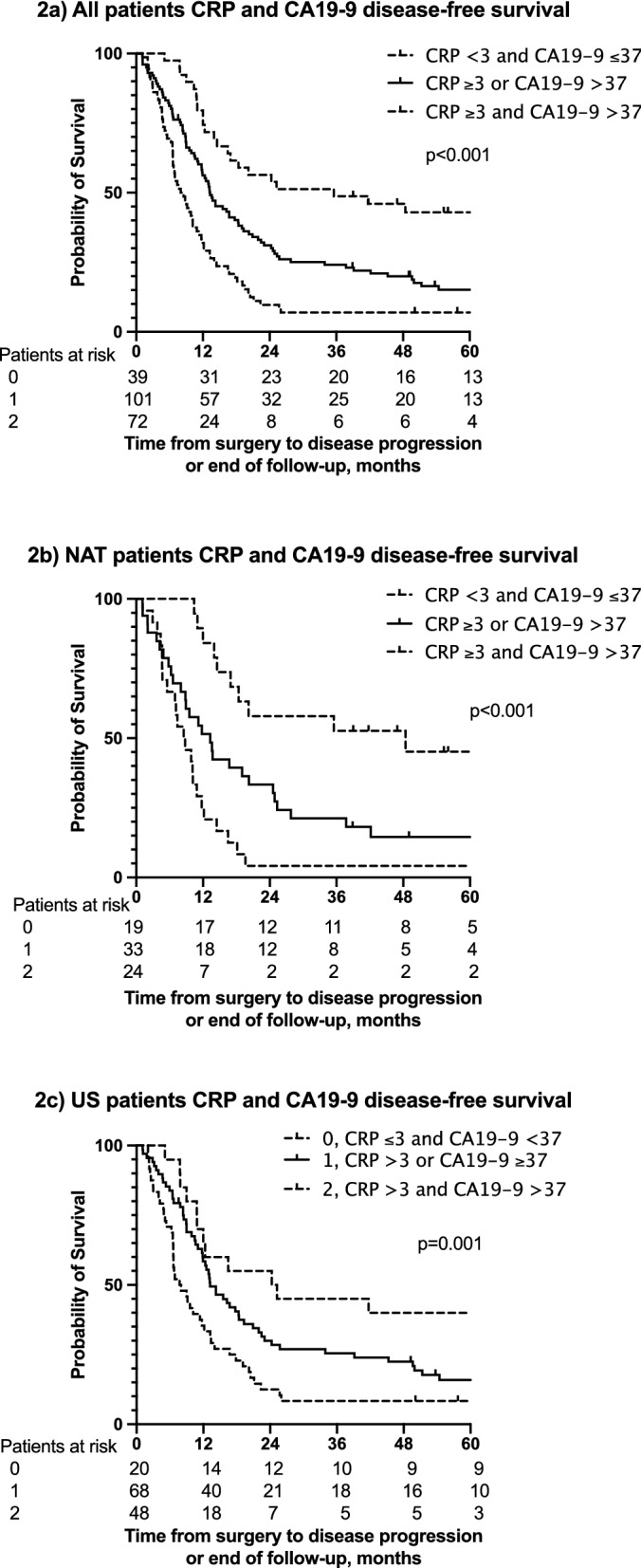
(**a**) Disease-free survival (DFS) for all patients according to CRP and CA19-9 levels. Median DFS were 36 m (95% CI 3–68 m), 13 m (95% CI 10–16 m), and 8 m (95% CI 6–10 m). (**b**) DFS for NAT patients according to CRP and CA19-9 levels. The median DFS were 48 m (95% CI –), 13 m (95% CI 9–18 m), and 9 m (95% CI 5–12 m). (**c**) DFS for US patients according to CRP and CA19-9 levels. The median DFS were 24 m (95% CI 5–44 m), 13 m (95% CI 9–17 m), and 8 m (95% CI 5–11 m).

CRP and CA19-9 were combined into a prognostic score: both CRP and CA19-9 below the cut-off values (3 mg/l and 37 kU/l, respectively), either CRP or CA19-9 above the cut-off value, and finally, both CRP and CA19-9 above the cut-off values. Among all patients, the corresponding median disease-specific survival times were 54 months (n = 39; 95% CI could not be calculated), 27 months (n = 101; 95% CI 22–32 months) and 16 months (n = 72; 95% CI 12–21 months, *p* < 0.001; Fig. 1a), respectively. For disease-free survival, we observed a similar pattern: 36 months (95% CI 3–68 months), 13 months (95% CI 10–16 months) and 8 months (95% CI 6–10 months; *p* < 0.001; Fig. 2a), respectively. At 5 years, among patients with CRP and CA19-9 levels below the cut-off values, 49% were alive and 45% were disease-free. These patients presented with the following characteristics: 21 patients presented at stage IA to IIA and 18 at stage IIB to III; the tumor was well-differentiated in 8, moderately in 21 and poorly in 7 patients (data were missing for 3 patients); radical resection was reached in 34 of 37 cases (data were missing for 2 patients); 20 were NAT patients and 19 underwent upfront surgery. Among NAT patients, if both the CRP and CA19-9 levels were below the cut-off value (n = 19), 52% were alive and 45% disease-free at 5 years. The median DSS has not been reached yet (Figs. 1b and 2b; Supplementary Table 2). Among patients undergoing upfront surgery, the median DSS was 46 months (n = 20; 95% CI 25–67 months); 45% were alive and 40% disease-free at 5 years (Figs. 1c and 2c; Supplementary Table 2). Adding albumin into the prognostic score did not improve the results (data not shown). However, all but one patient with CRP and CA19-9 levels below the cut-off values exhibited normal albumin levels (≥ 35 g/l).

### CRP and other laboratory values

**Figure 3:**
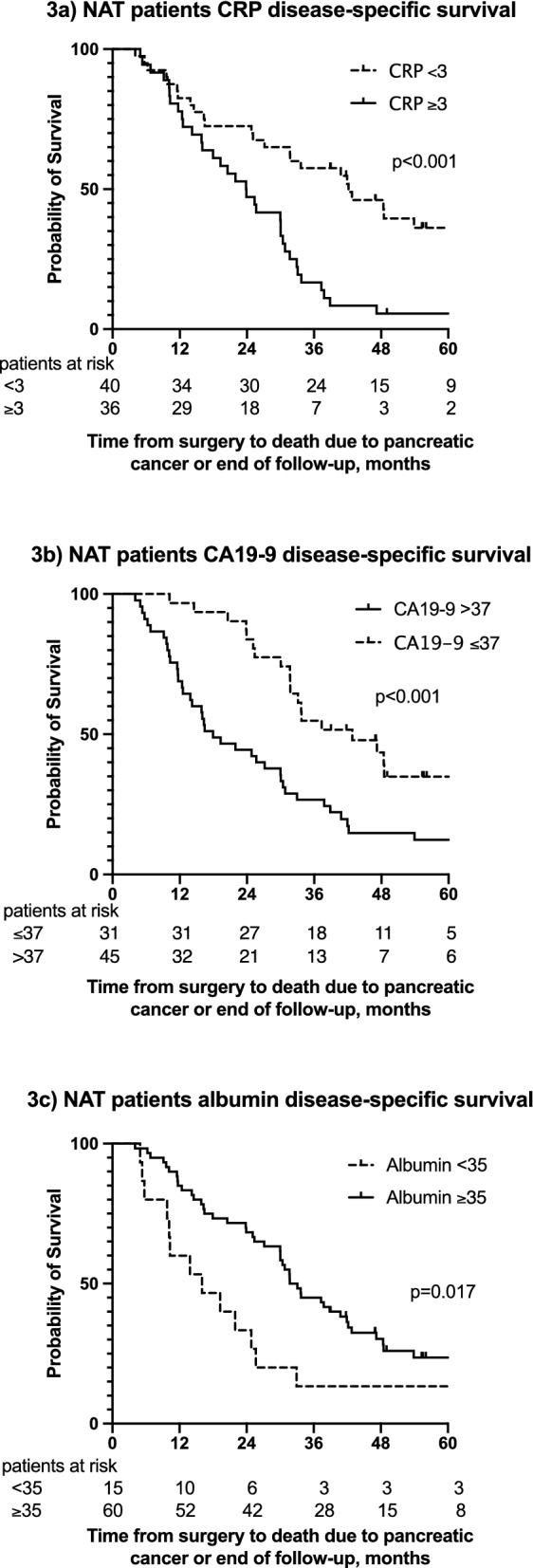
(**a**) Disease-specific survival (DSS) for NAT patients according to CRP levels. Median DSS were 42 m (95% CI 26–58 m) and 24 months (95% CI 17–31 months). (**b**) DSS for NAT patients according to CA19-9 levels. Median DSS were 43 m (95% CI 26–59 m) and 18 m (95% CI 10–26 m). (**c**) DSS for NAT patients according to albumin levels. Median DSS were 32 m (95% CI 28–35 m) and 16 m (95% CI 5–27 m).

Among NAT patients, CRP, CA19-9, albumin, CEA, GPS, and mGPS associated with DSS and DFS (Fig. 3; Supplementary Fig. 1; Supplementary Table 2). Among patients undergoing upfront surgery, CRP, CA19-9, bilirubin, and GPS associated with DSS, although only CRP and CA19-9 associated with DFS (Supplementary Figs. 2 and 3; Supplementary Table 2). With the used cut-off values, platelets, bilirubin, or leukocytes did not associate with DSS or DFS (Supplementary Table 2).

### Multivariate analysis

Among NAT patients, CRP and CA19-9 combined [either above the cut-off value hazard ratio (HR) 3.140, *p* = 0.019; both above the cut-off values HR 3.962, *p* = 0.013] predicted a poor outcome in our multivariate analysis (Table 3). Additionally, among NAT patients, the administration of adjuvant therapy predicted a favorable outcome (HR 0.428, *p* = 0.007; Table 3). Among patients undergoing upfront surgery, stage IIB-III (HR 3.379, *p* < 0.001), adjuvant therapy (HR 0.354, *p* < 0.001), perivascular invasion (HR 2.208, *p* = 0.002), and CRP and CA19-9 combined (either above the cut-off value HR 4.124, *p* = 0.001; both above the cut-off values HR 3.3480, *p* = 0.003) independently emerged as prognostic factors in our multivariate analysis (Table 3).

**Table 3:** Univariate and multivariate analysis for NAT patients and those undergoing upfront surgery separately.

NAT patients	Univariate		Multivariate	
	HR (95% CI)	*p *value	HR (95% CI)	*p *value
Age at operation < 65 y	0.994 (0.596–1.658)	0.982	1.072 (0.559–2.056)	0.833
Sex, male	0.986 (0.596–1.693)	0.986	0.997 (0.535–1.857)	0.993
Stage, IIB-III vs IA-IIA	1.321 (0.788–2.216)	0.291	0.932 (0.469–1.854)	0.842
Adjuvant therapy	**0.432 (0.255–0.732)**	**0.002**	**0.428 (0.231–0.792)**	**0.007**
Perivascular invasion	1.482 (0.864–2.543)	0.153	1.006 (0.466–2.171)	0.987
Perineural invasion	1.774 (0.960–3.281)	0.067		
Vascular resection	0.889 (0.530–1.494)	0.657		
Radical resection	**0.516 (0.283–0.940)**	**0.031**	0.608 (0.272–1.360)	0.226
Tumor differentiation, well	ref	0.754		
Moderately	1.130 (0.575–2.222)	0.723		
Poorly	1.373 (0.593–3.176)	0.459		
Albumin, < 35 g/l	**2.105 (1.127–3.932)**	**0.020**	1.342 (0.598–3.009)	0.475
CRP and CA19-9*, 1	**ref**	** < 0.001**	**ref**	**0.033**
2	**3.545 (1.607–7.818)**	**0.002**	**3.140 (1.212–8.138)**	**0.019**
3	**6.637 (2.897–15.207)**	** < 0.001**	**3.962 (1.330–11.804)**	**0.013**
**US patients**				
Age at operation, < 65 y	0.873 (0.599–1.272)	0.481	0.786 (0.491–1.259)	0.316
Sex, male	0.717 (0.488–1.055)	0.092	0.796 (0.502–1.262)	0.332
Stage, IIB-III vs IA-IIA	**2.334 (1.479–3.682)**	** < 0.001**	**3.379 (1.838–6.212)**	** < 0.001**
Adjuvant therapy	**0.668 (0.449–0.994)**	**0.046**	**0.354 (0.214–0.586)**	** < 0.001**
Perivascular invasion	**2.299 (1.547–3.415)**	** < 0.001**	**2.208 (1.353–3.604)**	**0.002**
Perineural invasion	0.914 (0.594–1.407)	0.683		
Vascular resection	1.085 (0.715–1.647)	0.702		
Radical resection	0.688 (0.434–1.090)	0.111	1.224 (0.705–2.125)	0.472
Tumor differentiation, well	**ref**	**0.002**		
Moderately	1.095 (0.647–1.852)	0.735		
Poorly	**2.421 (1.322–4.435)**	**0.004**		
Albumin, < 35 g/l	1.512 (0.963–2.374)	0.072	1.200 (0.702–2.048)	0.505
CRP and CA19-9*, 1	**ref**	**0.001**	**ref**	**0.002**
2	**2.103 (1.096–4.037)**	**0.025**	**4.124 (1.857–9.162)**	**0.001**
3	**3.306 (1.684–6.491)**	**0.001**	**3.348 (1.488–7.532)**	**0.003**

The end-point was disease-specific death. *CRP and CA19-9: (1) both CRP and CA19-9 below cut-off value (3 and 37, respectively), (2) either CRP or CA19-9 above the cut-off value, and (3) both values above the cut-off value. The multivariate model was stratified by grade.

*NAT* neoadjuvant therapy, *US* upfront surgery.

### Laboratory values based on patient- and tumor-related factors

NAT patients with disease recurrence or death due to pancreatic cancer within 12 months postoperatively (n = 37) exhibited higher CRP (*p* = 0.002) and CA19-9 levels (*p* < 0.001) than patients with no recurrence (Supplementary Table 3). Additionally, those who survived for more than 5 years postoperatively (n = 9) exhibited lower CRP (*p* = 0.004) levels (Supplementary Table 3). Only one NAT patient with preoperative CRP ≥ 3 (total, n = 36) survived for more than 5 years (CRP = 3.93). Among patients undergoing upfront surgery, those with recurrence within 12 months (n = 67) exhibited lower albumin (*p* = 0.005) and higher CA19-9 levels (*p* = 0.021), and those surviving for more than 5 years postoperatively (n = 25) exhibited higher albumin (*p* < 0.001) and lower CA19-9 levels (*p* = 0.001; Supplementary Table 4).

## Discussion

In this retrospective study, we demonstrate that combining CRP and CA19-9 in a prognostic score predicts postoperative outcomes in PDAC patients. A 5-year survival rate of 20–25% has been previously reported among resected PDAC patients^2,3^. However, in the present study, we identified a subgroup of NAT patients with a 5-year survival rate of 52% and a subgroup of patients undergoing upfront surgery with 5-year survival rate of 44%. These subgroups were characterized by low CRP and CA19-9 levels irrespective of disease stage and tumor differentiation. Additionally, combining CRP and CA19-9 differentiated patients with a poor survival. To the best of our knowledge, this represents the first study combining CRP and CA19-9 levels into a prognostic score. The primary advantages of this prognostic score are the preoperative availability of CRP and CA19-9, in addition to their affordable price. CRP and CA19-9 represent routine markers and no other marker with this level of accessibility has previously been reported to predict survival like the combination of CRP and CA19-9 in this study.

Resulting from an inflammatory state, the liver increases CRP production^16^; elevated CRP levels reflect the systemic inflammatory response. Cancer is known to cause an inflammatory response, which associates with factors affecting survival, such as cachexia, fatigue, malnutrition, and a slower clearance of anticancer drugs and, thus, higher treatment-related toxicity^12^. Additionally, high preoperative CRP and CA19-9 levels could indicate micrometastatic disease, predict inadequate postoperative recovery, and interruptions or poor delivery of adjuvant therapy, resulting in a poor prognosis. Therefore, combining CRP and CA19-9 was logical and reasonable. Moreover, NAT appears to affect the tumor and its microenvironment, and the cancer-related inflammatory response^25^. Based on these aspects we hypothesized that the examined markers impact the survival of NAT patients and those undergoing upfront surgery differently. The results of this study support our hypothesis. Both CRP alone and CRP and CA19-9 combined differentiated long-term survivors better among NAT patients than those undergoing upfront surgery.

Many previous reports indicated the prognostic value of CRP in advanced PDAC patients receiving palliative treatment with cut-off values ranging from 1 to 20 mg/l^18,19,21–24^. Similarly, CRP levels have correlated with tumor burden, indicating the aggressiveness of advanced pancreatic cancer^25^. In resected PDAC, without a separate analysis of NAT patients, high CRP associated with a poor prognosis: a median survival of 21 (CRP ≤ 3 mg/l) versus 13 months in 51 patients (CRP > 3 mg/l)^20^ and median survival times of 32 months (CRP ≤ 5 mg/l), 24 months (CRP > 5–15 mg/l) and 14 months (CRP > 15 mg/l)^17^ in 256 patients were reported. In another study on 474 patients (27% resected), a low CRP (< 4.5 mg/l) associated with a good prognosis^22^. Our results agree with these findings. Additionally, we managed to demonstrate the prognostic value of CRP in NAT patients and compare NAT patients to patients who underwent upfront surgery in a quite large cohort of resected patients. In contrast, Pine et al.^19^ found that a high CRP (> 5 mg/l) did not associate with outcome in resected patients (n = 58) and Garcea et al.^29^ found no correlation between CRP levels and disease recurrence (n = 74).

With our high-sensitivity method, we successfully explored the effect of low-grade inflammation on PDAC survival with slightly elevated levels of CRP. The results obtained by our high-sensitivity CRP method are comparable with standard CRP assays, however, the high-sensitive method is able to measure levels of even < 1 mg/l. In our study, we were able to show a correlation between slightly elevated CRP levels—as a marker of low-grade inflammation—and survival. In fact, among NAT patients, the median CRP of patients with disease recurrence within 12 months (4.4 mg/l) was only slightly higher than the reference limit (3 mg/l) routinely used in Finnish laboratories. Furthermore, in GPS and mGPS a CRP level of 4.4 mg/l would represent a low value. We must note that only one NAT patient with CRP ≥ 3 mg/l survived for more than 5 years, thereby emphasizing the role of inflammation in cancer survival. The CRP level of this patient was 3.93 mg/l. Interestingly, this agrees with previous results showing a correlation between low-grade inflammation and an increased risk of cardiovascular risk death^30^.

Different prognostic scores combining CRP and albumin (GPS, mGPS, and the CRP-to-albumin ratio), have been shown to predict survival in PDAC^31–35^. A median survival of 17 months has been reported for a GPS score of 2, 26 months for a score of 1, and 28 months for a score of 0; for mGPS, median survival reached 17, 28, and 26 months, respectively, for the scores of 2, 1, 0^35^. Our simple prognostic score combining CRP and CA19-9 proved superior to these prognostic scores, with a median survival of up to 54 months. In our study, a low preoperative albumin level associated with poor postoperative survival among NAT patients but did not reach statistical significance among patients who underwent upfront surgery. Combining albumin to our prognostic score along with CRP and CA19-9 did not prove feasible nor improve our results.

Previous PDAC studies on CA19-9 demonstrated that patients with low levels of CA19-9 (< 37 kU/l) had a longer median survival (32–36 months) than those with high levels (> 37 kU/l, 12–15 months)^36^. Additionally, a preoperative CA19-9 > 100 kU/l predicted early recurrence (at 6 months) and overall a poor postoperative prognosis^37^. These findings agree with ours, although in our study both NAT patients and those undergoing upfront surgery with CA19-9 ≤ 37 kU/l exhibited a longer median survival (NAT 43 months; upfront surgery 46 months).

Because 5–10% of the Caucasian population cannot synthesize CA19-9, we could not determine whether patients with CA19-9 ≤ 37 kU/l had low values resulting from the cancer biology or due to Lewis antigen-negativity. This represents one limitation of CA19-9 and thereby of our study. However, patients with undetectable CA19-9 (levels below 2 kU/l) appear to experience a similar survival to those with levels ≤ 37 kU/l, and a better prognosis than those with elevated levels^38^. Other limitations to our study include the lack of high-sensitivity CRP determined before NAT, the limited number of NAT patients, and the lack of a validation cohort. The fact that only operated patients were included in the study might cause bias, however, the main purpose of the study was to find preoperative prognostic factors. We additionally acknowledge the fact that the retrospective nature of the study might cause selection bias. However, the strengths of our study include the ability to compare NAT patients to those undergoing upfront surgery and to determine low levels of CRP using the high-sensitivity CRP assay. The main strength is that we could demonstrate the positive prognostic value of a low CRP level in addition to the negative prognostic value of an elevated CRP level and that we were able to introduce a new prognostic score for PDAC.

The results of this study show that the combination of two preoperative prognostic biomarkers provides an essential improvement in the preoperative evaluation of the clinical outcome of PDAC patients. Interestingly, CA19-9 is a typical PDAC biomarker whereas CRP is thought to reflect the patient’s systemic reaction to the tumor. Thus, the prognosis of PDAC appears to rely on both tumor- and patient-related factors. Even a low-grade systemic inflammation reflected by CRP had a significant impact on patient prognosis. The wide availability and low cost of these laboratory tests render them valuable in the preoperative evaluation of PDAC patients. Considering the overall poor prognosis of PDAC, even among those resected, the extraordinary prognosis of patients with a low CRP in combination with a low CA19-9 should be noted. These markers have not been studied together before, making this finding notable. Investigating CRP and CA19-9 on operated NAT patients is especially interesting, adding new valuable information to pancreatic cancer research. This new and simple prognostic score deserves to be further validated in other patient cohorts.

## Supplementary information

Supplementary figures.

Supplementary tables.

## Data Availability

The data analyzed and generated within the study are not publicly available because they contain data that have not been published as such yet.
