# Supplementary material for: Combining CRP and CA19-9 in a novel prognostic score in pancreatic ductal adenocarcinoma
Source: Sci Rep. 2021 Jan 12;11:781. doi: 10.1038/s41598-020-80778-0 (PMC7804300; doi:10.1038/s41598-020-80778-0)

TITLE PAGE

Combining CRP and CA19-9 in a Novel Prognostic Score in Pancreatic Ductal Adenocarcinoma

Running title: A novel pancreatic cancer prognostic score

Anna M. (AM) Nurmi[a], Harri K. (HK) Mustonen[a], Ulf-Håkan (UH) Stenman[b], Hanna E. (HE) Seppänen[a,c*], Caj H. (CH) Haglund[a,c*]

*these authors contributed equally to this work

[a]*Department of Surgery, Helsinki University Hospital, University of Helsinki, PO Box 22, 00014 University of Helsinki, Helsinki, Finland*

[b]*Department of Clinical Chemistry, Helsinki University Hospital, Haartmaninkatu 8 PO Box 63, 00014 University of Helsinki, Helsinki, Finland*

[c]*Translational Cancer Medicine Research Program, Faculty of Medicine, Haartmaninkatu 8 PO Box 63, 00014 University of Helsinki, Helsinki, Finland*

Corresponding author: Anna M. Nurmi, Department of Surgery, University of Helsinki, Helsinki University Hospital, Finland, PO Box 22, 00014 University of Helsinki, Finland, E-mail:anna-maria.nurmi@helsinki.fi, Tel. +358 50 427 0042

Supplementary Figure 1a) Disease-free survival (DFS) for NAT patients according to CRP levels. The median DFS were 20 months (95% CI 11-30 months) and 10 months (95% CI 8-12 months).

Supplementary Figure 1b) DFS for NAT patients according to CA19-9 levels. The median DFS were 18 months (95% CI 7-30 months) and 9 months (95% CI 5-13 months).

Supplementary Figure 1c) DFS for NAT patients according to albumin levels. The median DFS were 15 months (95% CI 10-19 months) and 7 months (95% CI 4-10 months).

Supplementary Figure 2a) Disease-specific survival (DSS) for patients undergoing upfront surgery according to CRP levels. Median DSS were 30 months (95% CI 22-38 months) and 23 months (95% CI 17-30 months).

Supplementary Figure 2b) DSS for patients undergoing upfront surgery according to CA19-9 levels. Median DSS were 46 months (95% CI 24-68 months) and 21 months (95% CI 15-27 months).

Supplementary Figure 2c) DSS for patients undergoing upfront surgery according to albumin levels. Median DSS were 26 months (95% CI 20-33 months) and 23 months (95% CI 14-32 months).

Supplementary Figure 3a) Disease-free survival (DFS) for patients undergoing upfront surgery according to CRP levels. The median DFS were 13 months (95% CI 9-17 months) and 12 months (95% CI 8-16 months).

Supplementary Figure 3b) DFS for patients undergoing upfront surgery according to CA19-9 levels. The median DFS were 22 months (95% CI 13-32 months) and 10 months (95% CI 8-12 months).

Supplementary Figure 3c) DFS for patients undergoing upfront surgery according to albumin levels. The median DFS were 12 months (95% CI 9-15 months) and 12 months (95% CI 10-14 months).

1a) NAT patients CRP disease-free survival

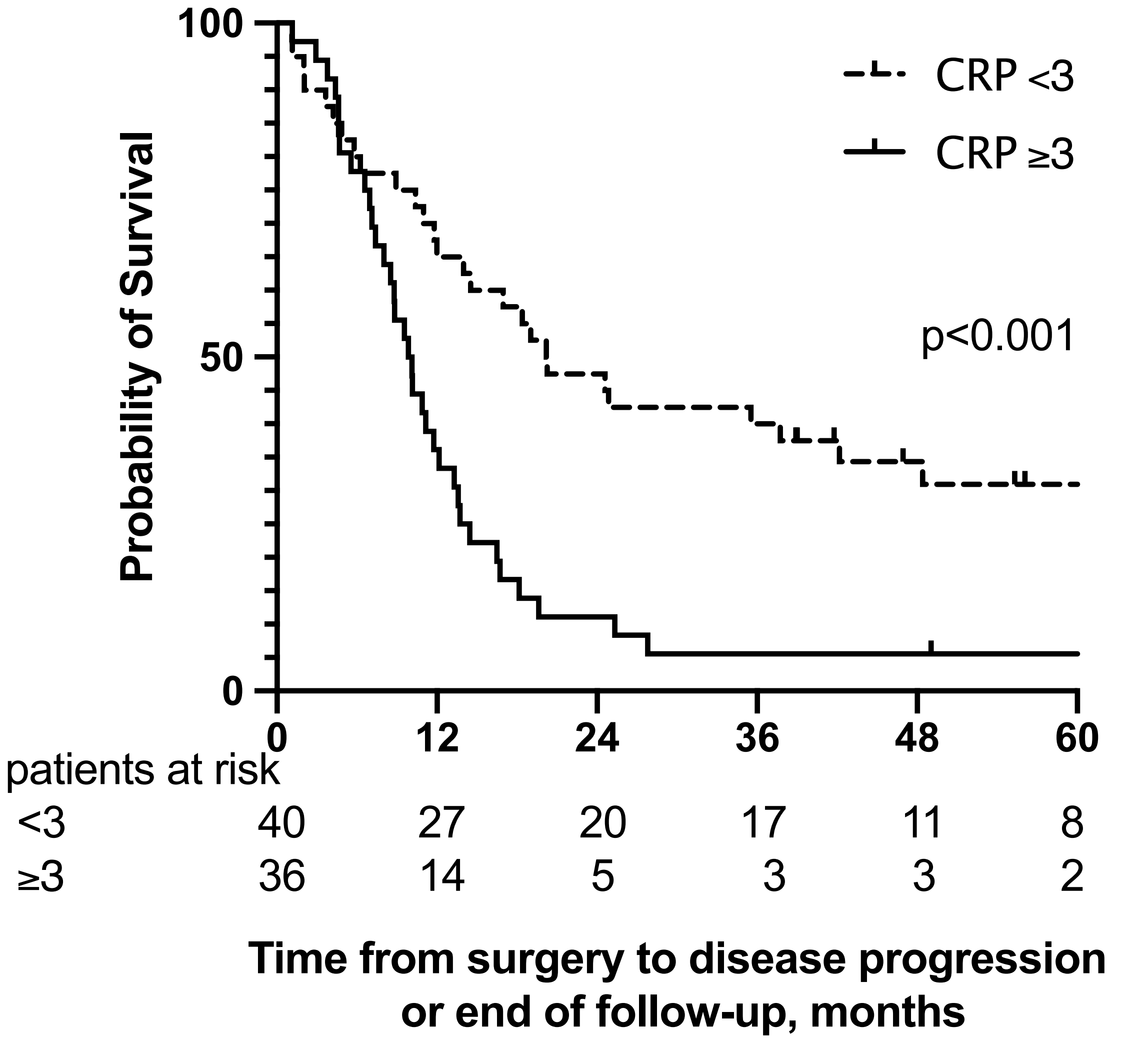

1b) NAT patients CA19-9 disease-free survival

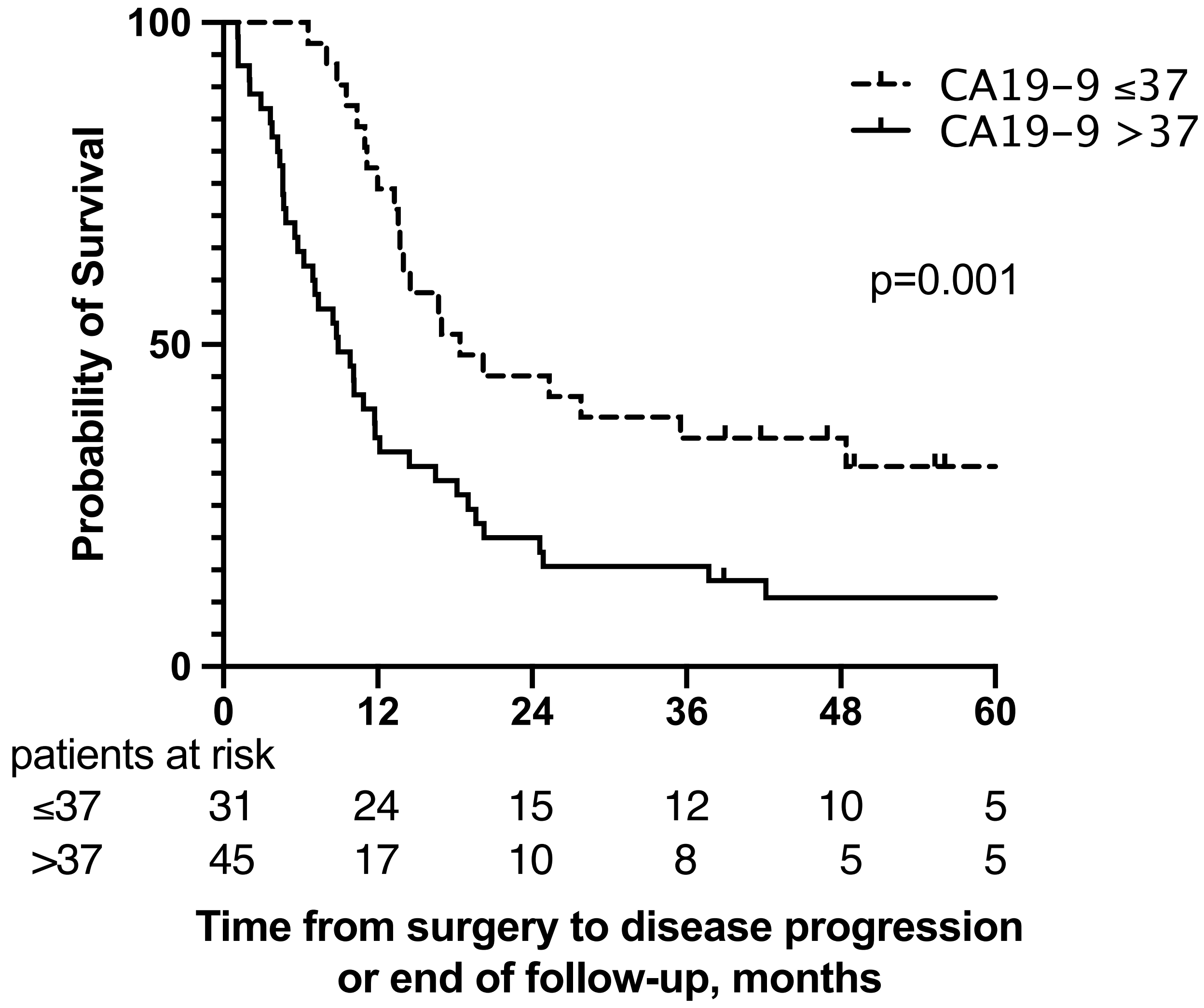

1c) NAT patients albumin disease-free survival

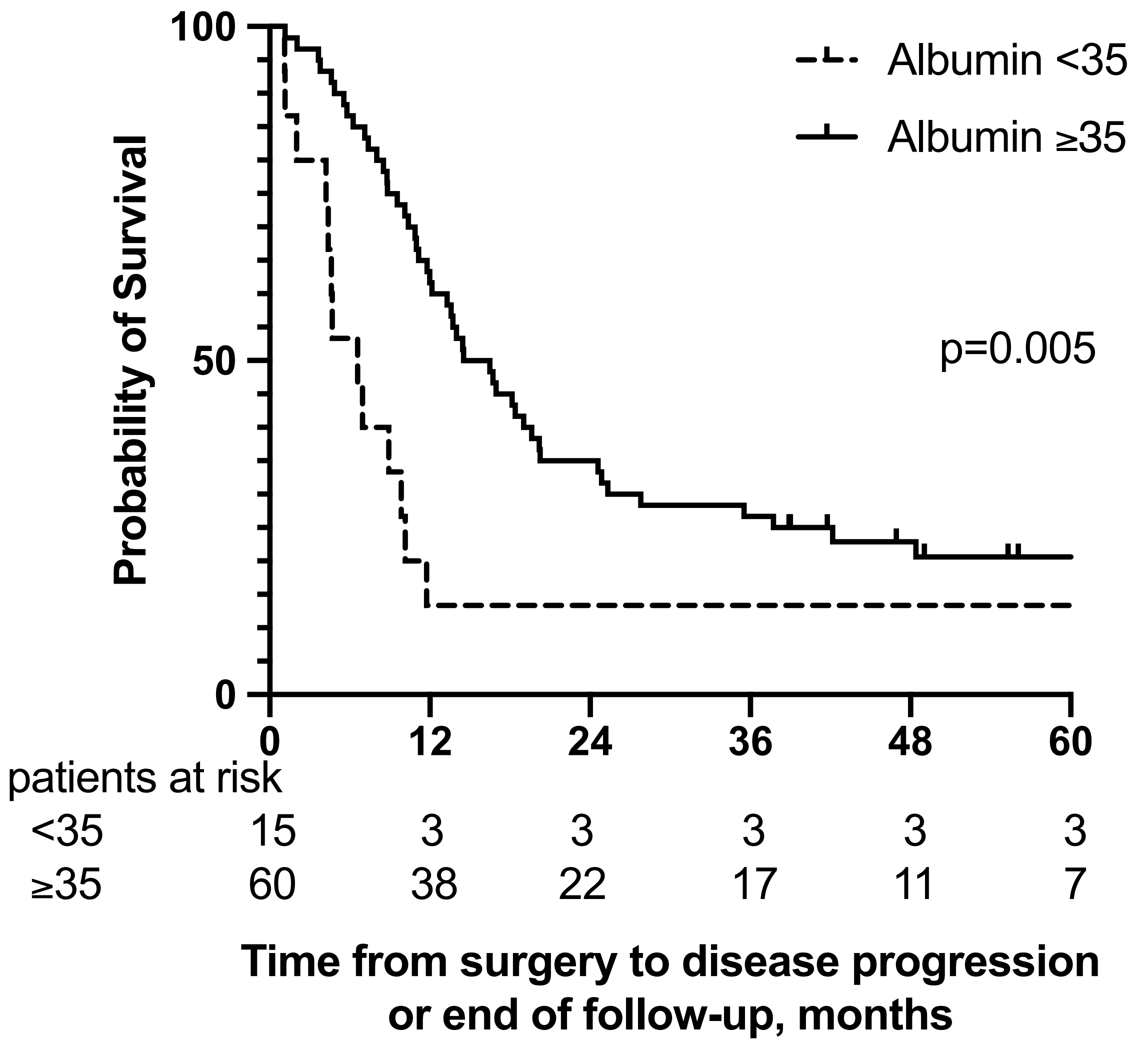

2a) US patients CRP disease-specific survival

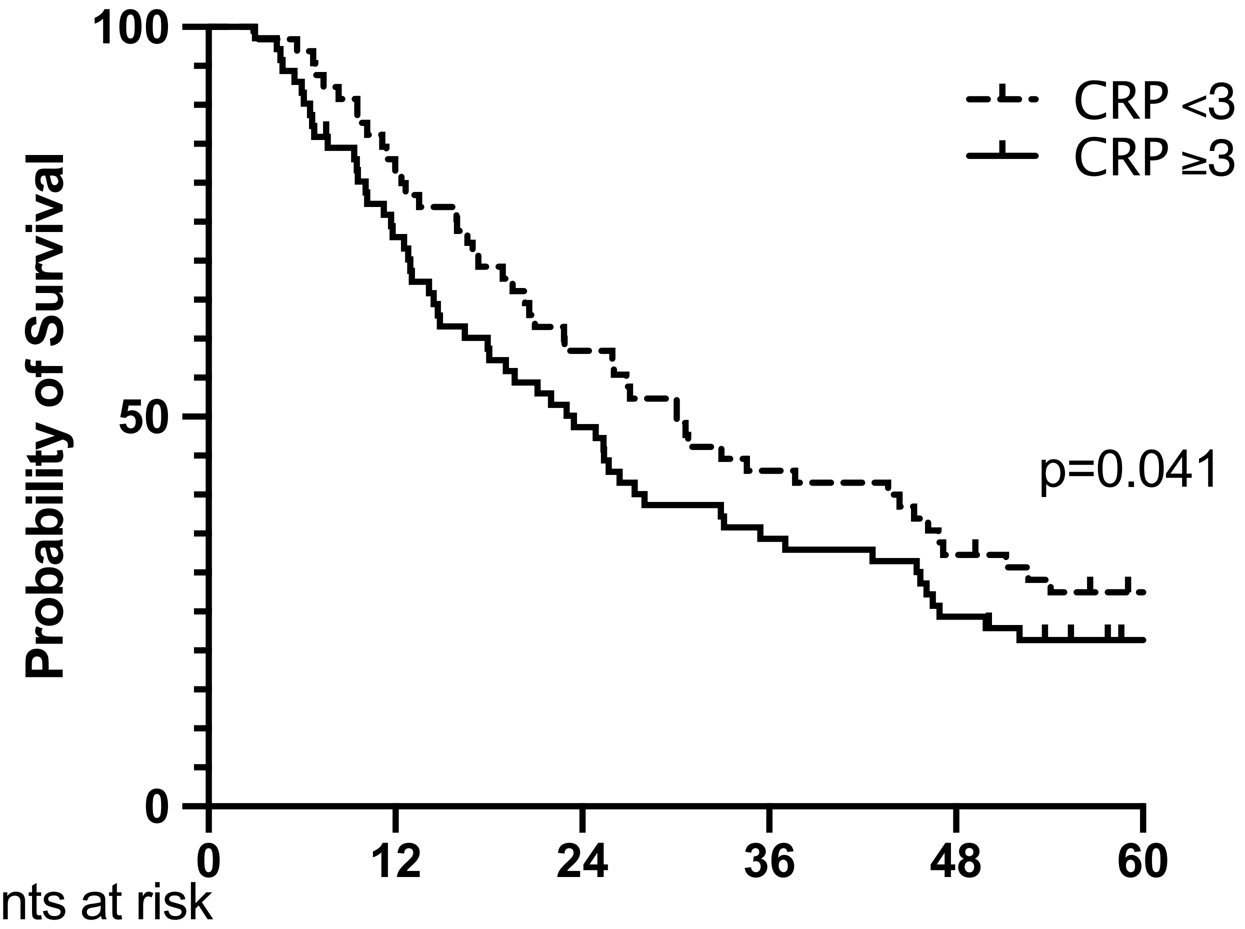

patients at risk						
<3	65	54	39	29	22	16
≥3	71	52	35	25	18	11

Time from surgery to death due to pancreatic cancer or end of follow-up, months

2b) US patients CA19-9 disease-specific survival

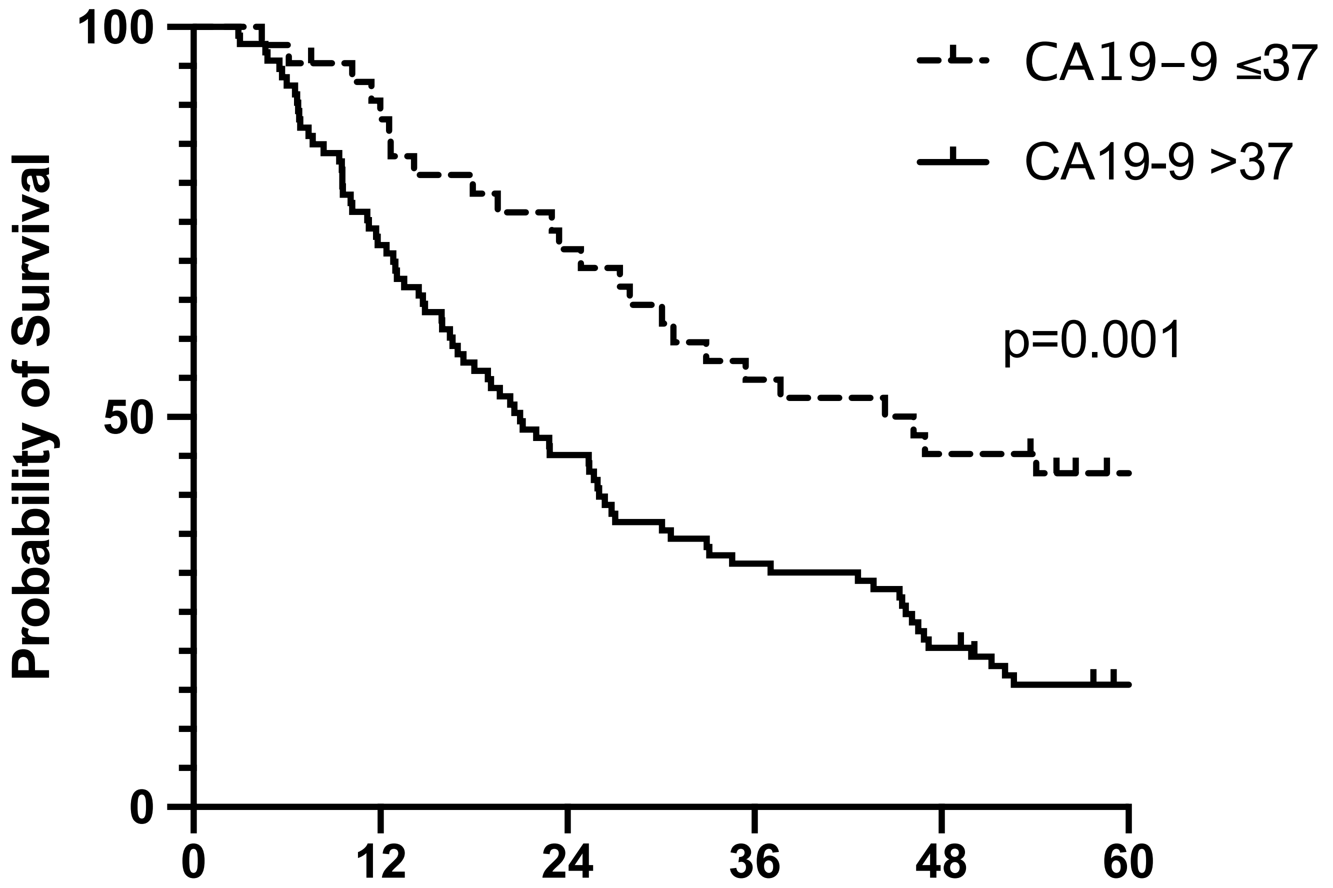

patients at risk						
≤37	43	38	31	24	20	15
>37	93	68	43	30	20	12

Time from surgery to death due to pancreatic cancer or end of follow-up, months

2c) US patients albumin disease-specific survival

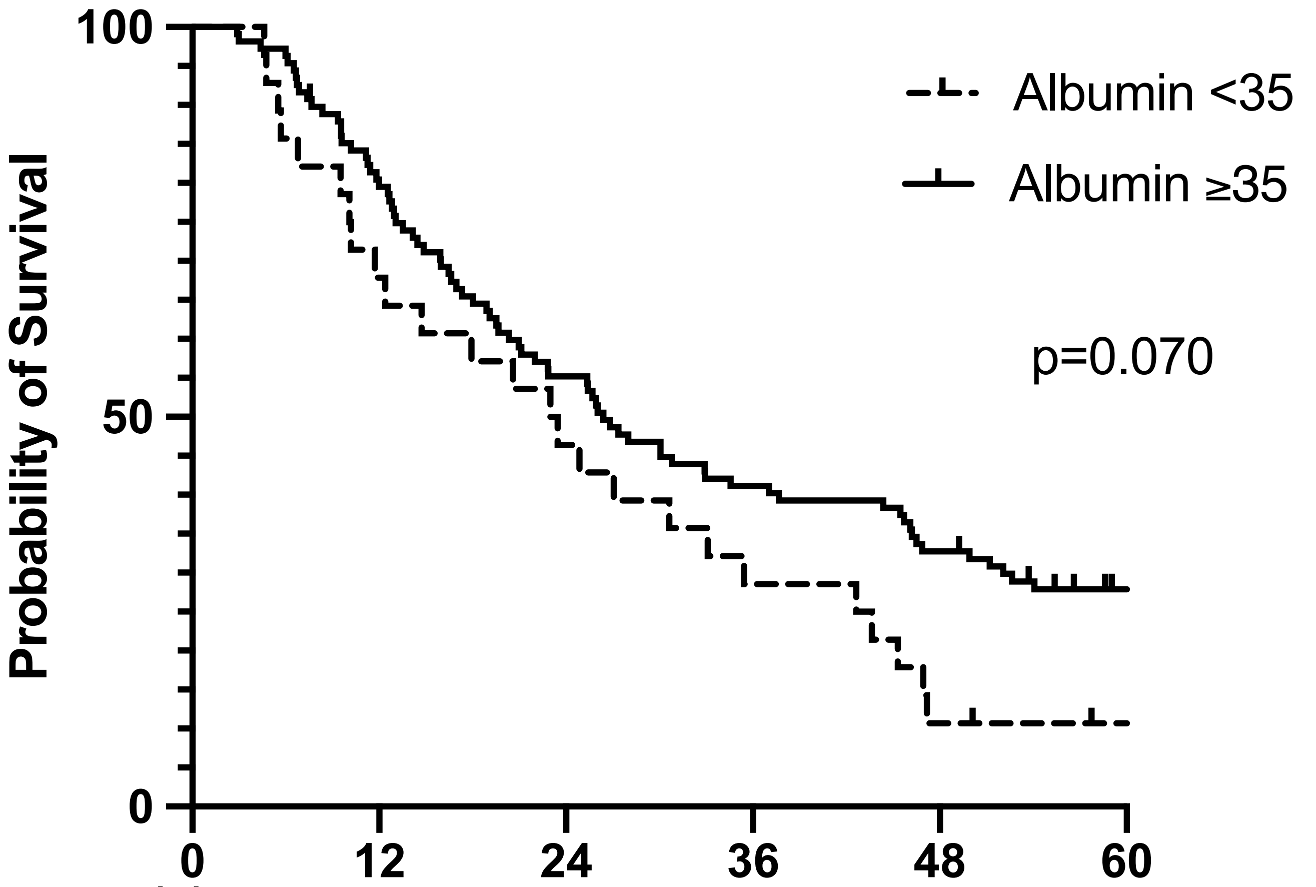

patients at risk						
<35	28	20	14	9	4	2
≥35	108	86	60	45	36	25

Time from surgery to death due to pancreatic cancer or end of follow-up, months

3a) US patients CRP disease-free survival

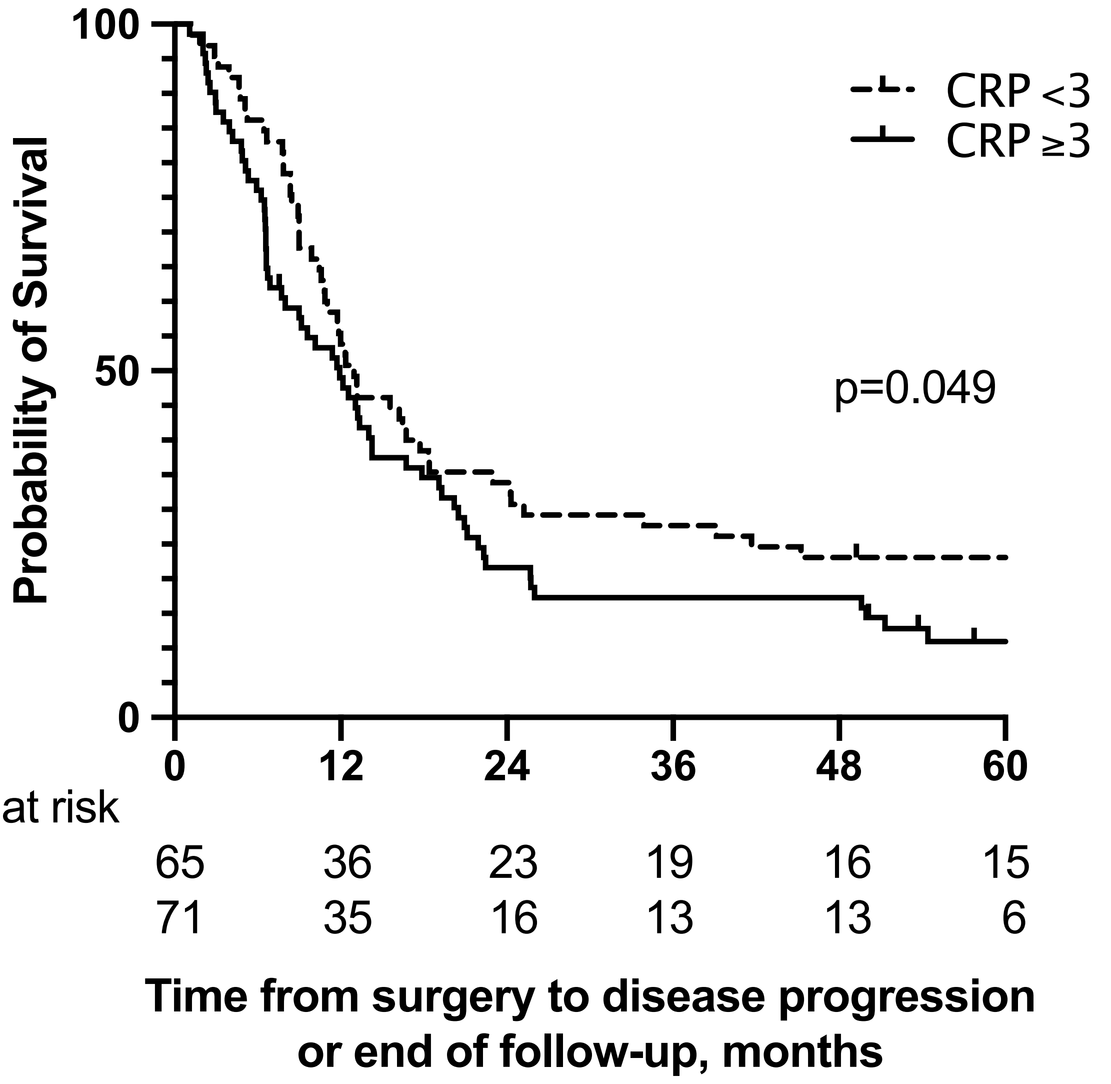

3b) US patients CA19-9 disease-free survival

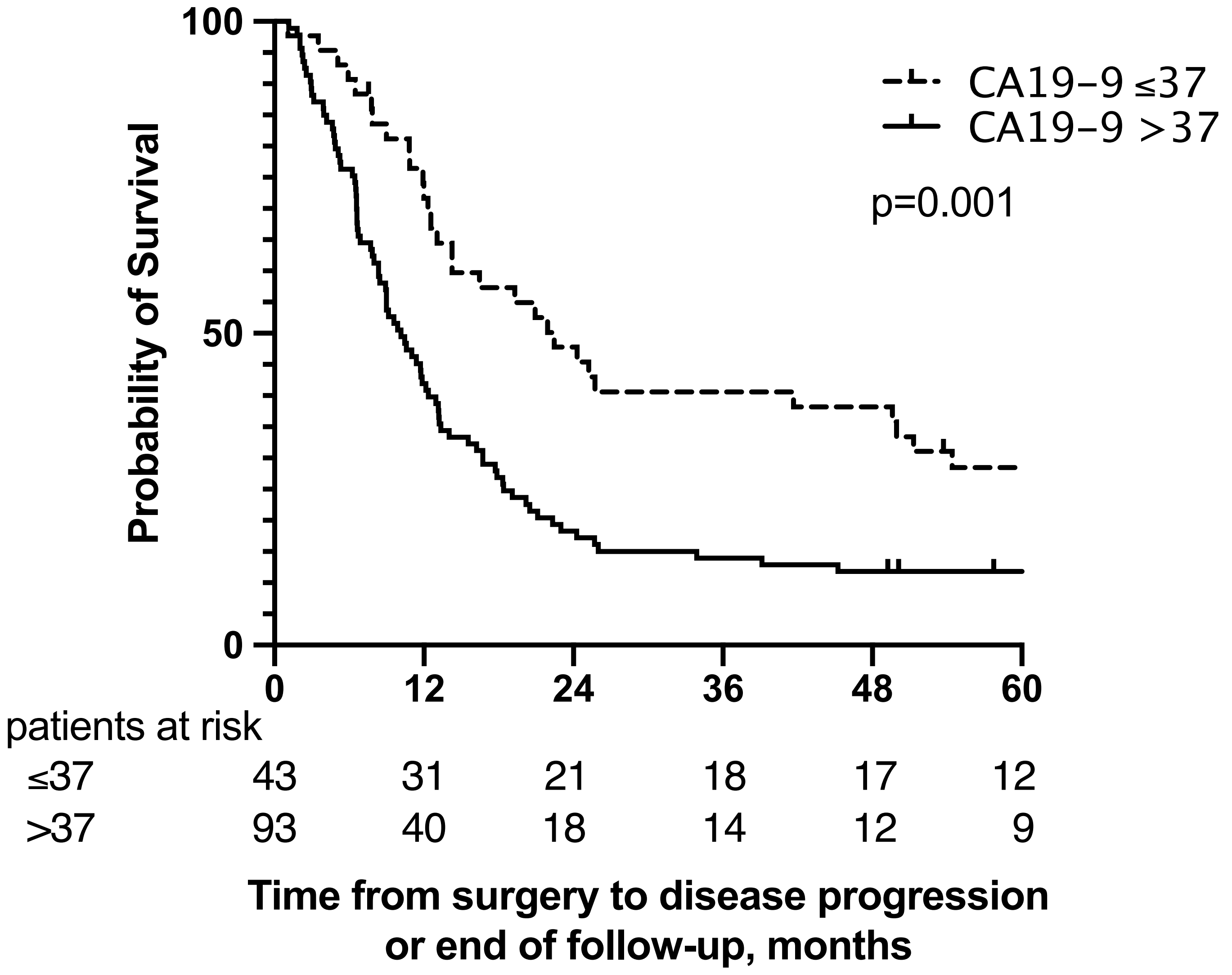

3c) US patients albumin disease-free survival

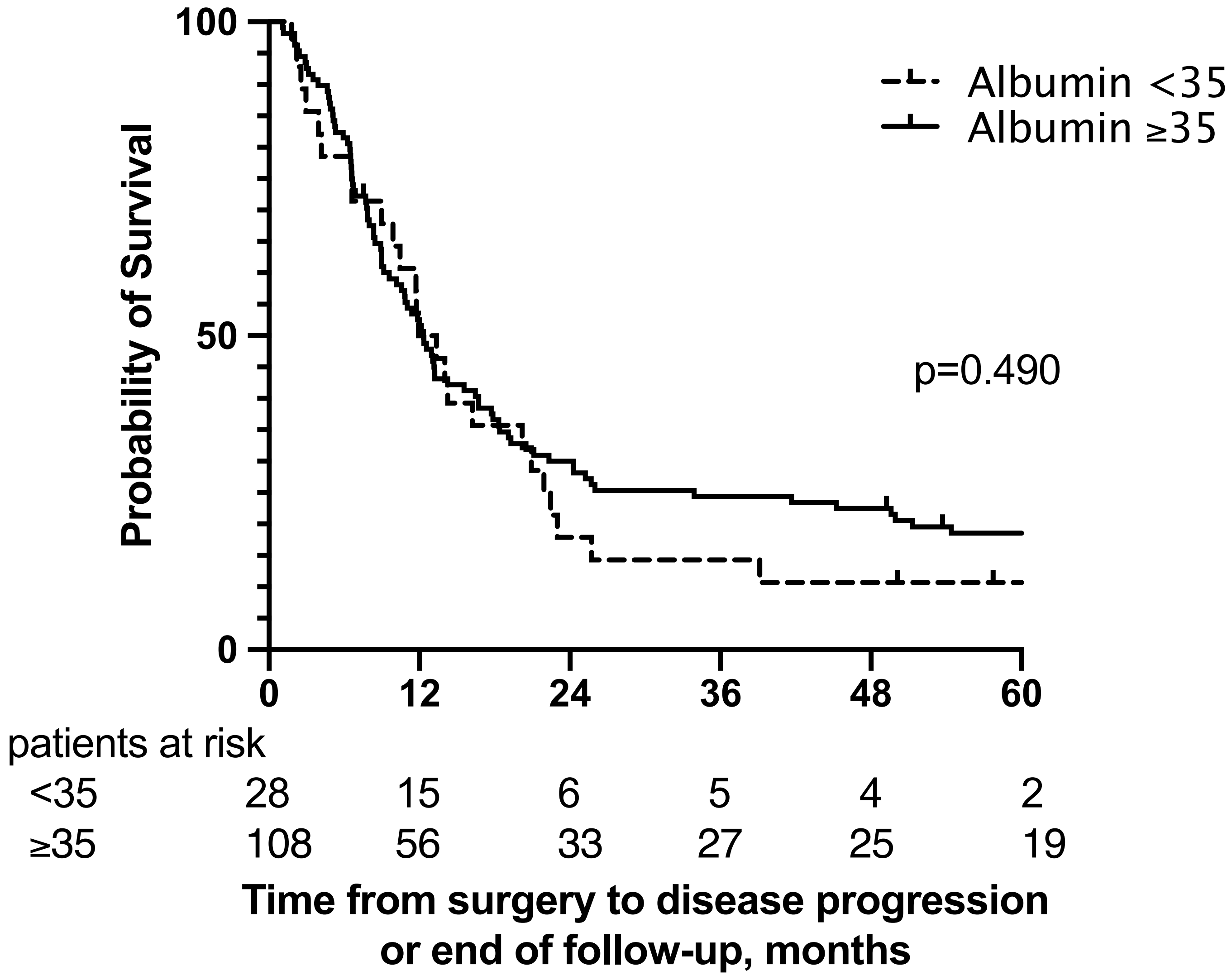

Supplement: Supplementary file 1 — Supplementary figures. [file 41598_2020_80778_MOESM1_ESM.pdf]
