# Supplementary material for: Combining CRP and CA19-9 in a novel prognostic score in pancreatic ductal adenocarcinoma
Source: Sci Rep. 2021 Jan 12;11:781. doi: 10.1038/s41598-020-80778-0 (PMC7804300; doi:10.1038/s41598-020-80778-0)
Supplement: Supplementary file 2 — Supplementary tables. [file 41598_2020_80778_MOESM2_ESM.pdf]

Supplementary Table 1. All neoadjuvant therapy and adjuvant therapy regimens listed in detail. Adjuvant therapy regimens separately for NAT patients and those undergoing upfront surgery.

**NAT patients – Neoadjuvant therapy regimens (n=76)**	**US patients – Adjuvant therapy regimens (n=94)**
8 x Gemcitabine 3-8 cycles	83 x Gemcitabine 1-6 cycles
3 x Gemcitabine 3-4 cycles, and gemcitabine chemoradiotherapy 50,4 Gy	4 x Gemcitabine chemoradiotherapy
2 x Gemcitabine 1 cycle, Nab-paclitaxel + gemcitabine 2 cycles	1 x Gemcitabine 1 cycle, changed to capecitabine 2 cycles
1 x Gemcitabine 1 cycle, gemcitabine + cisplatin 7 cycles, gemcitabine 2 cycles	2 x Gemcitabine + nab-paclitaxel 6-8 cycles
9 x Gemcitabine chemoradiotherapy 50,4 Gy	4 x Capecitabine 3-7 cycles
1 x Gemcitabine chemoradiotherapy 50,4 Gy, and gemcitabine + cisplatin 4 cycles	
25 x Gemcitabine + cisplatin 3-7 cycles	
6 x Gemcitabine + cisplatin 3-6 cycles, and gemcitabine chemoradiotherapy 45-60Gy	
4 x Gemcitabine + cisplatin 3-7 cycles, and capecitabine chemoradiotherapy 50,4Gy	
2 x Gemcitabine + capecitabine 3-5 cycles	
3 x Gemcitabine + nab-paclitaxel 3-4 cycles	
8 x Folfirinox 5-9 cycles	
1 x Folfirinox 5 cycles and Stereotactic body radiation therapy	
1 x Folfirinox 3 cycles, and Gemcitabine + nab-paclitaxel 3 cycles	
2 x not specified	
**NAT patients – Adjuvant therapy regimens (n=52)**	
36 x Gemcitabine 1-8 cycles	
1 x Gemcitabine 1 cycle, changed to cabecitabine 3 cycles	
8 x Gemcitabine + cisplatin 3-4 cycles	
1 x Gemcitabine + cisplatin 1 cycle, reduced to gemcitabine 2-3 cycles	
2 x Gemcitabine + capecitabine 2-3 cycles	
1 x Gemcitabine + nab-paclitaxel 3 cycles	
2 x Capecitabine 5-8 cycles	
1 x Folfirinox 7 cycles	

NAT = neoadjuvant therapy, US = upfront surgery, Gy=Gray

Supplementary Table 2. Median survival times (disease-specific and disease-free, Kaplan-Meier analysis) for NAT patients and those undergoing upfront surgery according to the preoperative markers. Units: CEA (≤5 vs >5.1 μg/l), platelets (<150 vs 150-360 vs >360 E9/l), bilirubin (≤20 vs >20 μmol/l), leukocytes (<3.4 vs 3.4-8.2 vs >8.2 E9/l) and for GPS and mGPS 0, 1 and 2. DSS = disease-specific death, DFS = disease-free survival, NAT = neoadjuvant therapy, US = upfront surgery.

Factors	NAT (95% CI), months	*P* = Value	US (95% CI), months	*P* = Value
**CRP**				
DSS	42 (26-58) vs 24 (17-31)	**<0.001**	30 (22-38) vs 23 (17-30)	**0.041**
DFS	20 (11-30) vs 10 (8-12)	**<0.001**	13 (9-17) vs 12 (8-16)	**0.049**
**CA19-9**				
DSS	43 (26-59) vs 18 (10-26)	**<0.001**	46 (24-68) vs 21 (15-27)	**0.001**
DFS	18 (7-30) vs 9 (5-13)	**0.001**	22 (13-32) vs 10 (8-12)	**0.001**
**CRP and CA19-9**				
DSS	- (-) vs 27 (18-36) vs 16 (10-22)	**<0.001**	46 (25-67) vs 27 (21-33) vs 16 (11-22)	**0.001**
DFS	48 (-) vs 13 (9-18) vs 9 (5-12)	**<0.001**	24 (5-44) vs 13 (9-17) vs 8 (5-11)	**0.001**
**Albumin**				
DSS	32 (28-35) vs 16 (5-27)	**0.017**	26 (20-33) vs 23 (14-32)	0.070
DFS	15 (10-19) vs 7 (4-10)	**0.005**	12 (9-15) vs 12 (10-14)	0.490
**CEA**				
DSS	32 (28-35) vs 14 (9-18)	**0.014**	26 (20-32) vs 25 (6-45)	0.129
DFS	14 (9-19) vs 6 (2-10)	**0.038**	13 (11-14) vs 11 (5-17)	0.418
**Platelets**				
DSS	19 (12-26) vs 30 (23-37) vs 39 (18-60)	0.247	45 (14-76) vs 26 (22-30) vs 20 (0-42)	0.772
DFS	10 (3-18) vs 14 (11-17) vs 20 (0-41)	0.435	17 (7-26) vs 12 (10-14) vs 11 (2-19)	0.664
**Bilirubin**				
DSS	30 (25-36) vs 16 (-)	0.421	27 (19-36) vs 21 (14-27)	**0.048**
DFS	14 (10-17) vs 5 (-)	0.147	12 (9-15) vs 12 (9-15)	0.279
**Leukocytes**				
DSS	15 (8-21) vs 32 (28-36) vs 22 (0-45)	0.414	17 (-) vs 25 (21-30) vs 33 (16-49)	0.919
DFS	11 (1-22) vs 14 (8-20) vs 12 (7-17)	0.792	12 (-) vs 12 (10-14) vs 13 (12-15)	0.884
**GPS**				
DSS	34 (26-41) vs 22 (15-29) vs 16 (3-29)	**0.013**	27 (21-34) vs 23 (13-34) vs 12 (0-34)	**0.049**
DFS	17 (11-23) vs 9 (4-14) vs 10 (3-17)	**0.007**	12 (10-15) vs 13 (9-17) vs 12 (3-21)	0.313

**mGPS**				
DSS	32 (24-39) vs 30 (30-30) vs 16 (3-29)	**0.043**	27 (22-32) vs 21 (8-35) vs 12 (0-34)	0.090
DFS	14 (8-20) vs 14 (13-14) vs 10 (3-17)	**0.043**	13 (10-15) vs 10 (2-18) vs 12 (3-21)	0.216

Supplementary Table 3. This table shows Preoperative biomarkers matched against patient- and tumor-related factors among NAT patients. *AJCC = American Joint Committee on Cancer 8th edition. **Patients the possibility of 5-year survival. Units: CRP mg/l, Albumin g/l, CA19-9 E9/l. DSS = disease-specific death, DFS = disease-free survival, NAT = neoadjuvant therapy. Mann-Whitney U test for groups of two and Jonckheere-Terpstra test for variables with more than two groups.

**NAT patients**	**CRP**		**CA19-9**		**Albumin**	
	**Median (IQR)**	***p***	**Median (IQR)**	***p***	**Median (IQR)**	***p***
**Sex**						
male (n=33)	2.1 (0.9-7.1)	0.626	104 (12-411)	0.801	37.6 (35.1-39.9)	0.801
female (n=43)	2.7 (1.5-4.9)		83 (15-375)		38.0 (36.0-40.5)	
**Age (years)**						
< 65 (n=37)	2.6 (0.9-6.2)	0.506	69 (17-422)	0.872	38.8 (36.3-40.0)	0.872
≥ 65 (n=39)	3.1 (1.3-5.6)		104 (15-366)		37.3 (34.8-40.0)	
**T***						
T0-T2 (n=69)	2.6 (1.1-5.3)	0.414	**87 (14-344)**	**0.045**	38.0 (36.0-40.1)	**0.045**
T3-T4 (n=7)	4.4 (1.7-9.4)		**250 (83-7 156)**		37.0 (35.0-38.0)	
**N***						
N0 (n=37)	2.9 (1.4-9.3)	0.399	**69 (10-291)**	**0.047**	38.0 (35.5-40.2)	**0.047**
N1 (n=26)	2.3 (1.1-5.2)		**91 (14-291)**		38.0 (36.4-40.4)	
N2 (n=13)	4.0 (0.7-5.5)		**682 (32-1 264)**		37.0 (35.5-39.7)	
**Stage***						
IA-IIA (n=37)	2.9 (1.4-9.3)	0.403	69 (10-291)	0.157	38.0 (35.5-40.2)	0.157
IIB-III (n=39)	2.6 (1.0-5.1)		133 (20-566)		37.8 (35.9-39.9)	
**Tumor size**						
≤30 mm (n=56)	2.7 (1.4-5.4)	0.967	**43 (11-318)**	**0.016**	**38.5 (36.1-40.4)**	**0.016**
>30mm (n=20)	2.5 (1.0-7.5)		**193 (85-940)**		**37.5 (33.3-38.0)**	
**Grade**						
1 (n=13)	2.3 (1.5-7.2)	0.973	30 (12-531)	0.311	38.0 (35.0-39.8)	0.311
2 (n=45)	2.7 (1.0-7.9)		87 (15-371)		37.9 (36.1-39.8)	
3 (n=14)	4.0 (1.2-4.7)		145 (57-823)		38.3 (34.7-40.8)	
**Cause of death**						
PDAC (n=53)	**4.0 (1.6-9.3)**	**<0.001**	**164 (28-624)**	**<0.001**	37.7 (34.9-39.7)	**<0.001**
other or alive (n=23)	**1.5 (0.9-2.6)**		**27 (4-92)**		38.0 (36.7-40.4)	
**DSS (months)**						
<12 (n=15)	4.0 (1.5-7.9)	0.118	**566 (215-1 124)**	**<0.001**	36.0 (34.0-39.7)	**<0.001**
12-24 (n=15)	4.5 (1.1-11.0)		**104 (31-250)**		37.0 (34.3-39.9)	
>24 (n=46)	1.9 (1.0-4.8)		**31 (10-167)**		38.2 (37.0-40.2)	
**DFS (months)**						
<12 (n=37)	**4.4 (1.6-8.6)**	**0.002**	**215 (58-693)**	**<0.001**	36.5 (34.1-39.5)	**<0.001**

12-24 (n=16)	**3.5 (1.1-10.6)**		**26 (11-387)**		39.4 (37.8-40.8)	
>24 (n=23)	**1.5 (0.9-2.6)**		**27 (6-90)**		38.0 (36.7-40.1)	
**5-year survival****						
yes (n=9)	**1.5 (0.9-2.4)**	**0.004**	92 (7-239)	0.434	39.8 (35.5-41.5)	0.434
no (n=41)	**4.9 (1.8-11.2)**		125 (24-377)		37.9 (35.3-40.5)	

Supplementary Table 4. This table shows Preoperative biomarkers matched against patient- and tumor-related factors among patients undergoing upfront surgery. *AJCC = American Joint Committee on Cancer 8th edition. **Patients with the possibility of 5-year survival. Units: CRP mg/l, Albumin g/l, CA19-9 E9/l. DSS = disease-specific death, DFS = disease-free survival, US = upfront surgery. Mann-Whitney U test for groups of two and Jonckheere-Terpstra test for variables with more than two groups.

**US patients**	**CRP**		**CA19-9**		**Albumin**	
	**Median (IQR)**	***p***	**Median (IQR)**	***p***	**Median (IQR)**	***p***
**Sex**						
male (n=59)	2.5 (1.4-9.0)	0.685	120 (11-563)	0.346	37.8 (34.2-40.4)	0.689
female (n=77)	3.4 (1.5-7.8)		134 (31-758)		38.0 (36.0-40.5)	
**Age (years)**						
<65 (n=69)	3.6 (1.4-13.0)	0.453	123 (16-533)	0.370	38.3 (35.5-40.7)	0.217
≥65 (n=67)	2.8 (1.6-6.3)		126 (32-753)		37.6 (34.8-40.3)	
**T***						
T0-T2 (n=111)	3.1 (1.4-7.9)	0.308	**96 (19-488)**	**0.001**	38.1 (35.4-40.5)	0.205
T3-T4 (n=25)	2.8 (2.0-13.4)		**503 (88-2 107)**		36.2 (34.2-40.2)	
**N***						
N0 (n=38)	2.5 (1.3-5.2)	0.083	**64 (25-296)**	**0.011**	39.1 (35.2-40.5)	0.197
N1 (n=59)	3.2 (1.3-8.6)		**124 (19-391)**		37.5 (35.7-40.4)	
N2 (n=39)	4.5 (1.8-14.1)		**486 (54-1 877)**		37.1 (34.4-40.5)	
**Stage***						
IA-IIA (n=38)	2.5 (1.3-5.2)	0.156	64 (25-296)	0.097	39.1 (35.2-40.5)	0.392
IIB-III (n=98)	3.4 (1.5-10.9)		172 (21-804)		37.7 (35.2-40.4)	
**Tumor size**						
≤30 mm (n=75)	**2.4 (1.2-6.8)**	**0.030**	**52 (11-299)**	**<0.001**	**39.2 (36.3-40.9)**	**0.006**
>30mm (n=61)	**4.0 (1.9-14.1)**		**366 (84-1 136)**		**36.7 (34.3-40.0)**	
**Grade**						
1 (n=24)	2.8 (1.5-7.3)	0.405	59 (20-232)	0.143	37.5 (35.1-40.2)	0.375
2 (n=81)	2.6 (1.4-8.6)		135 (24-712)		38.5 (35.5-41.0)	
3 (n=28)	3.8 (2.1-9.1)		220 (25-924)		36.4 (34.4-39.7)	
**Cause of death**						
PDAC (n=107)	3.5 (1.5-9.0)	0.097	**143 (30-788)**	**0.021**	**37.4 (35.0-40.4)**	**0.005**
other or alive (n=29)	2.4 (1.1-4.8)		**41 (7-280)**		**39.9 (37.8-41.5)**	
**DSS (months)**						
<12 (n=32)	**6.3 (2.2-17.4)**	**0.034**	**333 (54-2 263)**	**0.001**	**36.2 (34.5-39.4)**	**0.011**
12-24 (n=32)	**3.3 (1.5-6.7)**		**250 (50-926)**		**37.4 (35.2-41.0)**	
>24 (n=72)	**2.5 (1.4-6.7)**		**64 (16-327)**		**38.7 (36.6-40.7)**	
**DFS (months)**						
<12 (n=67)	3.6 (1.8-11.3)	0.097	**299 (55-1 110)**	**0.021**	**36.8 (35.1-40.0)**	**0.005**

12-24 (n=32)	3.6 (1.4-11.2)		**77 (20-318)**		**37.5 (34.6-40.3)**	
>24 (n=37)	2.3 (1.1-6.4)		**30 (6-205)**		**39.9 (37.7-41.3)**	
**5-year survival****						
yes (n=25)	2.5 (1.5-6.1)	0.231	**30 (6-129)**	**0.001**	**39.9 (38.0-42.0)**	**<0.001**
no (n=100)	3.4 (1.5-9.3)		**179 (48-855)**		**37.2 (34.9-40.0)**	
